# Ultrastructural Changes in Rat’s Atrial Cardiomyocytes After Short Term Administration of Amiodarone and Possible Protective Role of Vitamin E

**DOI:** 10.4021/jocmr909w

**Published:** 2012-05-15

**Authors:** Abdullah S. Shatoor, Mohamed Samir Ahmad Zaki, Refaat A. Eid, Mohamed A. Sayed-Ahmad

**Affiliations:** a Internal Medicine Department, Cardiology Section, Faculty of Medicine, King Khalid University, Saudi Arabia; b Anatomy Department, Faculty of Medicine, King Khalid University, Saudi Arabia; c Pathology Department (Electron Microscopy Unit), Faculty of Medicine, King Khalid University, Saudi Arabia

**Keywords:** Electron microscopy, Amiodarone, Cardiomyocytes

## Abstract

**Background:**

Amiodarone chlorhydrate is a diiodated benzofuran derivative used to treat a variety of cardiac rhythm abnormalities. The use of amiodarone is associated with ultrastructural changes affecting body tissues, but its effect on the ultrastructure of the heart has not yet been fully elucidated.

**Methods:**

The aim of this study is to test the adverse effects of amiodarone administration on cardiomyocytes and to study the possible protective role of vitamin E co-administration. A total of 18 adult male albino rats were used in this study. The rats were divided randomly into three groups of 6 rats each as follows: group I was considered the control group and was given vegetable oil; group II received 54 mg/kg of oral amiodarone; and group III received a single dose of combined vitamin E (50 mg/kg) and amiodarone (54 mg/kg). After 2 weeks, the rats were sacrificed, and the atrial tissues were harvested and processed for electron microscopic study.

**Results:**

Administration of amiodarone alone modified the atrial architecture, which was demonstrated by the following: mitochondrial enlargement and cristae lysis; marked heterogeneity of myofibril patterns with partial necrosis and disintegration of myofilaments; and irregularities of the sarcomere and less concentration of atrionatriuretic factor (ANF) granules, which localised in closed proximity to the nucleus with disrupted chromatin contents. Concomitant administration of vitamin E with amiodarone showed a considerable preservation of the atrial architecture.

**Conclusions:**

Oral administration of amiodarone in rats resulted in ultrastructural changes in atria, which can be attenuated by vitamin E co-administration.

## Introduction

It is well established that long-term oral administration of amiodarone is extremely effective in the management of most supraventricular and ventricular tachyarrhythmias [1, 2]. Amiodarone is categorised as a class III antiarrhythmic agent, as it prolongs the atrial and ventricular action potential duration (APD) as well as the refractory period of cardiac muscle. These effects are potentiated when the drug is given for longer durations [3].

Compared to the action of most available antiarrhythmic agents, the oral administration of amiodarone has a negative inotropic effect of lower significance and is less proarrhythmic, making it advantageous for use in patients with impaired ventricular function [4, 5]. On the contrary, intravenous administration of amiodarone in studies conducted on dogs resulted in considerable reduction of myocardial contractility [6, 7].

In spite of these advantages, adverse effects caused by amiodarone are frequent and can be serious and even lethal. Such reactions involve the liver [8], lungs [9-11], cornea [12], skin [13] and thyroid gland [14]. Evidence from in vitro and in vivo studies has attributed the pathogenesis of amiodarone toxicity to oxygen free radicals and oxidative stress [9, 10]. However, other studies do not support these findings. The effects of amiodarone on the mitochondrial oxidation of fatty acids in the liver of mice were determined [15-18]. Characteristic lamellar lysosomal inclusion bodies representing phospholipidosis were found in microscopically studied liver specimens [17].

Vitamin E (α-tocopherol) is thought to protect tissues by reducing or preventing oxidative damage. This lipid soluble vitamin prevents lipid peroxidation chain reactions in cellular membranes by interfering with the propagation of lipid radicals. Recent histological and immunohistochemical studies have shown a protective role of vitamin E against oxidative stress injury and alteration in the architecture of liver and lungs of rats that received amiodarone [19, 20].

Therefore, the aim of the present study is to assess the ultrastructural changes of the rat heart after amiodarone administration and to test the possible protective effect of vitamin E co-administration.

## Materials and Methods

### Chemical agents

Amiodarone (Chlorhydrate D’) was purchased from Sanofi (France), and alpha tocopherol acetate was purchased from Sigma-Aldrich (Buchs, Switzerland). Both compounds were used without further purification.

### Drug preparation

Amiodarone alone or combined with alpha tocopherol acetate were dissolved in 2 mL vegetable oil to their final concentration according to the weight of each animal. The doses of amiodarone and alpha tocopherol acetate used in this experiment were 54 mg/kg and 50 mg/kg, respectively. The dose of amiodarone selected in this study corresponds to the maximum human daily therapeutic dose converted into the equivalent rat dose according to Paget’s table [21]. The dose of alpha tocopherol was chosen as an effective antioxidant dose in rats according to Calfee-Mason et al [22].

### Experimental animals

The study was carried out in the Department of Anatomy at the College of Medicine, King Khalid University (Abha, Saudi Arabia) in October, 2011. Eighteen adult male albino Wistar rats weighing between 200 - 250 g and aged 6 months were used for the experimental procedure. The rats were obtained from the animal house of the College of Medicine, King Khalid University, Abha, Saudi Arabia. All rats were maintained in similar polypropylene cages of standard dimensions at a temperature of 25 ± 1^o^C and a standard 12 h day/night cycle; the rats were housed in groups of 4 rats per cage (50 x 26 x 16 cm). The rats were fed rat chow and water ad-libitum. All procedures were approved by the Ethical Committee at King Khalid University Medical School and were performed in agreement with the Principles of Laboratory Animal Care, advocated by the National Society of Medical Research and the Guide for the Care and Use of Laboratory Animals and published by the National Research Council [23].

### Experimental procedure

The rats were divided randomly into 3 groups of 6 rats each. All drugs were given in a final volume of 2 mL/day, orally using intra-gavage stainless steel needle for 2 weeks. The groups of rats were classified as follows: (a) the control group received vegetable oil; (b) group 2 received amiodarone (54 mg/kg) alone; and (c) group 3 received both amiodarone (54 mg/kg) and alpha tocopherol acetate (50 mg/kg).

### Preparation of atrial tissue for electron microscopy

After the treatment period, small pieces of the harvested atrial cardiac muscle were fixed in 2.5% glutaraldehyde for 24 hours and washed by phosphate buffer (0.1 M, pH 7.4). Post fixation was made in 1% osmium tetroxide buffered to pH 7.4 with 0.1 M phosphate buffer at 4 ^o^C for 1 - 2 hours. Specimens were washed again in phosphate buffer to remove excess fixative and dehydrated through ascending concentrations of ethanol. The specimens were then cleared in propylene oxide and embedded in araldite. Polymerisation was obtained by placing the capsules at 60 ^o^C. Ultra-thin sections (100 nm) were prepared using an ultra microtome and were placed on uncoated copper grids. Following double staining with uranyl acetate and lead citrate, sections were examined and photographed using a JEOL transmission electron microscope [24]. The specimens were prepared and read by a blinded histopathologist who was unaware of the different treatment groups.

## Results

### Group I (Control group)

**Figure 1:**
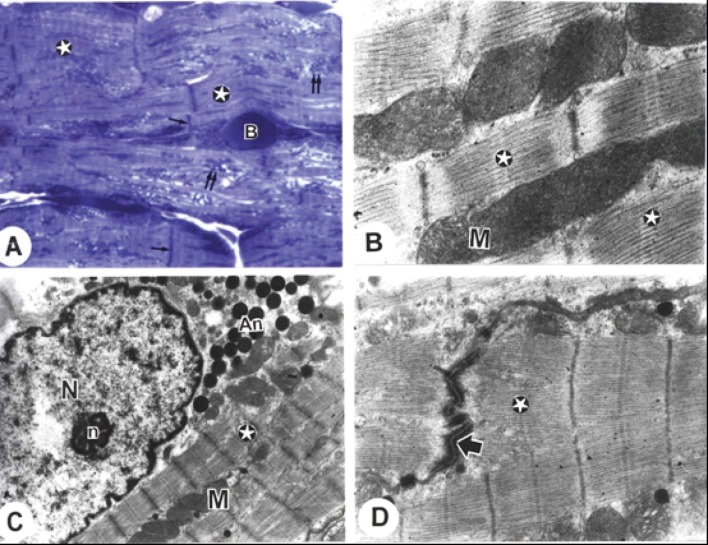
Electron micrographs from cardiac atria of control rats. A. A semithin section showing clarified intercalated discs (arrows), typical cross striations of muscle fibres (star), and many rows of mitochondria (double arrows). (B) blood capillary. B. The striation pattern (stars) with its typical alteration and dense mitochondria (M) rich in cristae (Bar = 200 nm). C. The striation pattern (stars) with its typical alteration and dense mitochondria (M) rich in cristae, euchromatic nucleus (N) with its nucleolus (n) and closely packed atrionatriuretic factor (ANF) granules (An) (Bar =1 µm). D. The striation pattern (stars) with its typical alteration and step-like intercalated disc (arrow) with adherent fascia (Bar = 500 nm). (x: 1000).

Electron microscopic examination of the sections from the rat atria of the control group showed normal architecture. The mitochondria were packed together between myofibrils with some beneath the sarcolemma and some around the nucleus with visible intercalated discs (Fig. 1A). The striation pattern, with its typical alteration and dense mitochondria rich in cristae, was clearly visible in cardiac muscle (Fig. 1B). The euchromatic nucleus with its nucleolus and closely packed large atrionatriuretic factor (ANF) granules were noted (Fig. 1C). Higher magnification of step-like intercalated discs was revealed tight junctions and desmosomes (Fig. 1D).

### Group II (Amiodarone treated rats)

**Figure 2:**
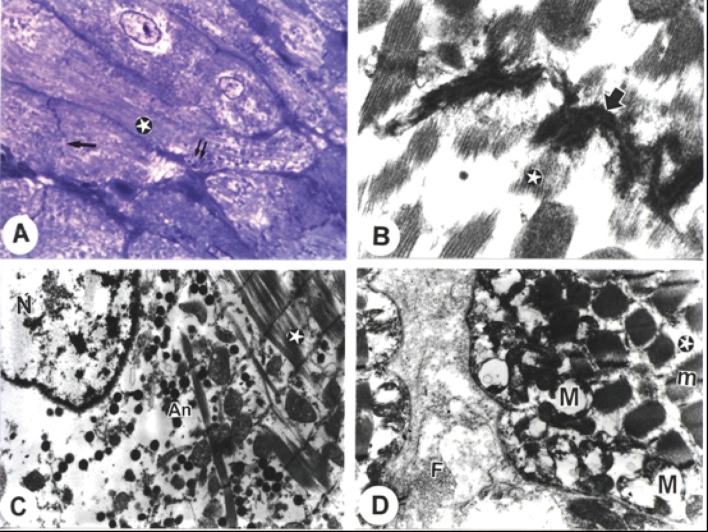
Electron micrographs from cardiac atria of amiodarone-treated rats. A. A semithin section showing abnormal pattern of striations (stars), disrupted intercalated discs (arrow) and abnormal mitochondrial arrangements (double arrows). B. Dissociation of myofibrils (stars) with irregularities and destruction of myofibrils and intercalated disc (arrow) (Bar = 200 nm). C. Dissociation of myofibrils (stars) with irregularities of the sarcomere and lesser concentration of ANF granules (An) with disrupted nuclear (N) chromatin contents (Bar = 1 µm). D. Swollen mitochondria (M) with loss of its cristae in some and loss of its pattern in others. Empty spaces in between myofibrils (m) and fibres (F) were noted in the intercellular space (Bar = 1 µm). (x: 1000).

Sections from atria of rats administered amiodarone alone, showed ultrastructural changes. Loss of myocardial organisation, a scattered area of degenerated cardiomyocytes, and myofibrillar disarray were apparent. Wavy myofibrils, abnormal pattern of striations, disrupted intercalated discs and abnormal mitochondrial arrangements were identified (Fig. 2A). Myofilaments derangement, partial necrosis, intercalated disc dissociation and disintegration of filaments were also observed (Fig. 2B). Irregularities of the sarcomere, lower concentration of ANF granules in the perinuclear area and disrupted nuclear chromatin were clearly observed (Fig. 2C). In addition, swollen mitochondria with lysis of its cristae and dissolution of the matrix were clearly observed. The intermembrane space of the mitochondria had expanded. In addition, specimens revealed decreased density of the sarcoplasm and marked heterogeneity in the myofibril pattern with ruptures and loss of continuity of the myofilaments (Fig. 2D).

### Group III (Amiodarone and alpha tocopherol treated rats)

**Figure 3:**
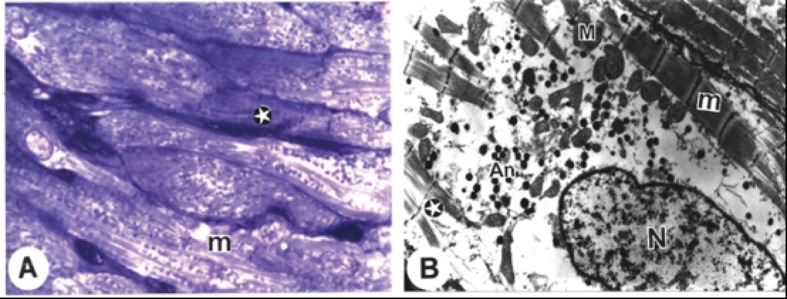
Electron micrographs from cardiac atria of amiodarone- and vitamin E-treated rats. A. A semithin section of cardiac muscle showing sarcoplasm of a cardiomyocyte occupied by normal myofibril striations (m), and other myofibrils (stars) appear disrupted. Dissociation of myofibrils in some (stars) and normal pattern (m) in others. B. ANF granules is still less in size (An) while the mitochondria (M) were preserved with its cristae (Bar = 1 µm). (x: 1000).

Concomitant administration of vitamin E with amiodarone showed a considerable protection of the atrial tissues. Microscopic study of the atrial tissue of this group showed that the sarcoplasm was occupied by normal myofibrils with clear striations and others had been disrupted. These changes did not demonstrate marked cardiac damage as in the amiodarone-treated group, although occasional focal loss of some myofibrils was observed (Fig. 3A, 3B). There were no alterations in mitochondrial morphology compared to the second group (preserved cristae, normal size and shape) (Fig. 3B).

## Discussion

Amiodarone produces its effect as an antiarrhythmic agent by employing different mechanisms of action. It is primarily a potassium channel blocker, but it can also block sodium and calcium channels in addition to beta- and alpha-adrenergic receptors [25]. The clinical use of amiodarone has been limited to refractory ventricular arrhythmias because of high incidence of side effects, which can be fatal [26]. Most cases of reported amiodarone cardiac toxicity are attributed to its electrophysiological effects, such as bradycardia, heart block and proarrhythmia, whereas the reported histopathological effects were limited mainly to non-cardiac tissues [27]. Hence, our current study is unique because it is the first in vivo study that demonstrates the ultrastructural changes due to amiodarone in its target cardiac cells.

Our study confirmed the toxic effects of amiodarone on mitochondrial morphology. The atrial specimens from the amiodarone-treated group showed swollen mitochondria with lysis of its cristae and dissolution of the matrices. The spaces between the outer and inner membranes of the mitochondria were clearly enlarged. Our findings support previous reported studies that showed that amiodarone induced perturbations and ultrastructural changes in mitochondrial structure of other non-cardiac tissues [28, 16]. The swelling of the mitochondria could result from failure of the osmotic control mechanism and/or decreased production of adenosine triphosphate with subsequent influx of water resulting in breakdown of the inner membranes [29-31]. Furthermore, it is possible that oxidative stress due to an increased amount of oxygen free radicals could cause oxidative damage to the mitochondria and release cytochrome C from mitochondrial intermembrane space, thus inducing apoptosis [32]. The reduced adenosine triphosphate production could also result in other changes, such as myofilaments derangement, partial necrosis, intercalated disc dissociation and disintegration of filaments along with irregularities of the sarcomere [31]. The alterations of the sarcolemma in cells with end-stage degeneration were associated with loss of myofibrils, loss of junctional contacts with adjacent cells and marked surface irregularity. Remodelling of the cell surfaces is an important feature of the cellular response to the stimuli of hypertrophy or degeneration [33]. Myofibril loss and sarcomere disarray are the most obvious structural changes in human cardiomyopathy and failing hearts [34].

The ANF-granules in the atria of rats treated with amiodarone alone were less concentrated and smaller in size. The enhancement of ANF synthesis in the cells to overcome cardiac overload, as in advanced heart failure, led to a reduction of the granule size with subsequent decrease in the levels of atrial ANF mRNA and plasma ANF in the course of the down regulation [35-37].

Concomitant administration of vitamin E with amiodarone, showed a considerable preservation of atrial architecture. The protective effects of vitamin E against ultrastructural changes in other tissues, such as those of the lung and liver, have been reported [38, 19, 20]. The possible mechanism of the protective effect of vitamin E on cardiomyocytes may be the result of several factors, including decreased cellular amiodarone accumulation, decreased fat rancidisation, increased membrane stabilisation, altered profibrotic gene expression, free radical scavenging and decreased apoptosis [39-42].

Although the protective benefit of vitamin E co-administration against amiodarone-induced cardiomyotoxicity is clearly observed in this report, the exact mechanism of this protection is not yet clear. The worrisome mechanism is that Vitamin E may negatively affect the antiarrhythmic efficacy of amiodarone. Furthermore, replicating the beneficial effects of vitamin E as antioxidant usually fails in clinical studies. The exact explanation for these conflicting results is not yet evident. However, Zoulin made a very interesting observation when he compared the results from animal and in vitro studies with results from human clinical trials. He found that the animals used in the antioxidative stress trials were already at abnormal levels of stress, whereas high-powered clinical trials lacked the confounding effect of stress and revealed no benefit or adverse outcome. However, smaller clinical studies involving patients with abnormal levels of stress, for example, studies involving diabetic patients, show some benefits. He concluded that antioxidant intervention during the so-called (WOPS) “Window Period for Oxidative Stress Attenuating Intervention” will show maximum benefit, whereas intervention outside this window will show no benefit or may even cause harm [43, 44]. Cardiac patients who require amiodarone therapy usually suffer from advanced heart disease, and their cardiac cells are expected to be under an abnormally high level of oxidative stress. Adding amiodarone to their drug regime, though effective in reducing arrhythmias, may lead to further progressive loss of myocardium and death due to pump failure rather than the arrhythmias themselves [45-47]. Therefore, it would be wise to consider adding antioxidant agents such as vitamin E when amiodarone use is contemplated.

### Conclusion

It is evident that amiodarone administered over a short period results in significant cardiac ultrastructural changes while concomitant administration of vitamin E provides some protection against these adverse effects. This finding raises a concern about the long-term use of amiodarone on the ultrastructure of the heart.
